# Efficacy of Communication Techniques and Health Outcomes of Bushfire Smoke Exposure: A Scoping Review

**DOI:** 10.3390/ijerph182010889

**Published:** 2021-10-16

**Authors:** Emily Heaney, Laura Hunter, Angus Clulow, Devin Bowles, Sotiris Vardoulakis

**Affiliations:** 1 ANU Medical School, Australian National University, Canberra, ACT 2601, Australia; Emily.Heaney@anu.edu.au (E.H.); Laura.Hunter@anu.edu.au (L.H.); Angus.Clulow@anu.edu.au (A.C.); 2 National Centre for Epidemiology and Population Health, Research School of Population Health, Australian National University, Canberra, ACT 2601, Australia; devin@atoda.org.au (D.B.); sotiris.vardoulakis@anu.edu.au (S.V.)

**Keywords:** bushfire, wildfire, smoke, air pollution, communication, media, public health, environmental health literacy

## Abstract

Public health officials communicate the relevant risks of bushfire smoke exposure and associated health protection measures to affected populations. Increasing global bushfire incidence in the context of climate change motivated this scoping review. English-language publications related to adverse health outcomes following bushfire smoke exposure and publications relating to communication during natural disasters were included. Bushfire smoke events potentially increase healthcare contact, especially presentations triggered by respiratory illness. At-risk populations include those with underlying cardiorespiratory disease, elderly, paediatric, pregnant persons, and First Nations people. We found that social media, television, and radio are among the most common information sources utilised in bushfire smoke events. Message style, content, and method of delivery can directly influence message uptake and behaviour modification. Age, rurality, and geographical location influence information source preferences. Culturally and linguistically diverse groups and those with hearing, vision, and mobility-related disabilities may benefit from targeted health recommendations. This review emphasises the health effects of bushfire smoke exposure and related communication recommendations during and after bushfire smoke events. Additional investigation may further clarify the health effects of bushfire smoke exposure and efficacy of related health messaging, particularly in at-risk populations. Quantitative comparison of communication methods may yield more specific recommendations for future bushfire smoke events.

## 1. Introduction

The Australian 2019–2020 bushfires burnt approximately 10 million hectares of land; more than the 2009 Black Saturday and 1983 Ash Wednesday bushfire disasters combined [1]. Outside of the devastating destruction of land, lives, and property, bushfire smoke was estimated to have affected more than 10 million Australians over a period of months, an unprecedented exposure period to bushfire smoke [2]. The North American and South American continents also experienced their own catastrophic bushfires in 2019–2020. Particulate matter smaller than 2.5 μm in diameter (PM_2.5_) generated by bushfires has been implicated by the World Health Organisation (WHO) as a potential causative factor in the development and exacerbation of cardiorespiratory diseases and cancers, triggering global concern that increasing incidences of exposure could result in a health emergency [3].

During bushfires, health and emergency service agencies are required to disseminate crucial health information in a timely manner or risk illness, disability, and loss of life [4]. The threat of increased morbidity and mortality during bushfire smoke events can be underappreciated when faced with imminent fire damage, but smoke potentially presents a threat in both short and long-term exposure [5]. Population subgroups who have pre-existing cardiorespiratory disease are likely at more significant risk [6]. As climate change triggers natural disasters with increasing frequency and intensity, it has never been more crucial to understand how to effectively communicate health information in times of crisis in order to most effectively minimise the impact on human health [7].

This scoping review aims to assess the current evidence regarding optimal public communication strategies used in smoke-related disaster scenarios. This review will inform the public health and emergency services on the best practices to connect with and empower populations to avoid exposure and potential health consequences associated with bushfire smoke. This study reviews publications covering the physical, mental, and psychosocial health outcomes of bushfire smoke exposure. It also discusses the effectiveness of communication techniques in reducing adverse health outcomes from bushfire smoke exposure and provides recommendations for health messaging in natural disasters.

## 2. Materials and Methods

### 2.1. Study Inclusion Criteria

**Figure 1:**
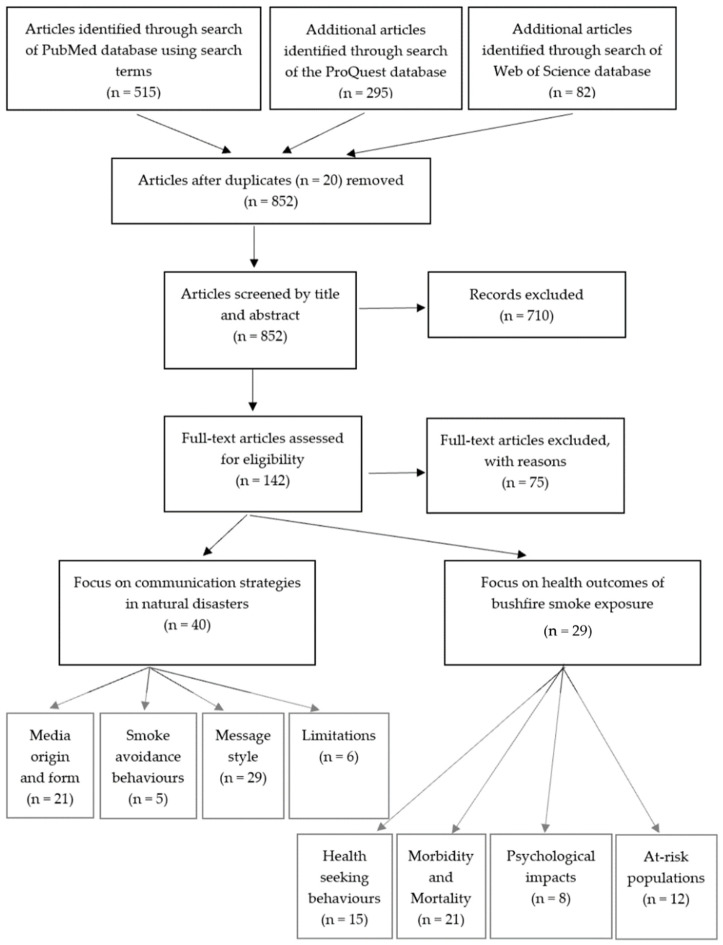
Selection of articles from the initial identification stage to abstract and full text screening before categorising into subsections for content analysis. Two of the included articles covered both communication strategies and health outcomes of bushfire smoke exposure.

This scoping review was conducted in accordance with the guidelines outlined by the Joanna Briggs Institute and summarised in Figure 1 [8]. Articles included contained information relating to the physical, mental, or psychosocial health outcomes of bushfire smoke. Articles detailing different communication techniques utilised to disseminate health warnings to at-risk subgroups and the general population during bushfires and other natural disasters were included. The included articles were required to be peer-reviewed, available online, and written in English.

Articles that focused on the health outcomes of firefighters and other first responders were excluded due to the confounding nature of the extreme smoke exposure experienced by these groups. Articles focussing on the health outcomes of smoke exposure from sources other than bushfires and wildfires, such as residential or industrial fires, were excluded as the nature of the particulate matter contained within the smoke may differ from that of bushfires. Articles focussing on the prediction and modelling of future bushfires, rather than on health outcomes or specific communication strategies associated with the events themselves, were also excluded. Full exclusion and inclusion criteria can be viewed in Supplementary Materials, Table S2.

### 2.2. Search Criteria

A primary search of the PubMed database was utilised to collect relevant articles. Our search parameters included terms such as ‘bushfire’, ‘prescribed burn’, and ‘health messaging’ (Supplementary Materials, Table S1). Other search terms included the use of MeSH terms including ‘wildfire’ and ‘social media’. A second search through the ProQuest and Web of Science databases was completed using similar search terms (Supplementary Materials, Table S1). Further articles were also collected by evaluating the reference lists of relevant systematic reviews. The search parameters included a 20-year published article time limit (1 January 2000 to 1 June 2020) to ensure relative recency of included articles, while still including any research motivated by previous events like the 2009 ‘Black Saturday’ bushfires in Australia.

### 2.3. Study Eligibility 

The initial collection of papers generated by the above search terms was evaluated independently by two members of the research team for relevance to the study inclusion criteria. The titles and abstracts of all articles were read to determine initial relevance with conflicting opinions resolved by a third member of the research team. Relevance was determined using predetermined inclusion and exclusion criteria (Supplementary Materials, Table S2) to reduce bias between different members of the research team. Articles that satisfied the inclusion criteria in this initial assessment were then re-assessed independently using the full text by two researchers, with disagreements resolved by a third member of the research team.

### 2.4. Data Extraction

Included papers were randomly assigned to two members of the research team to extract data from the publications. Extracted data were then reviewed by the third member of the research team. Data extracted included: study design, aim of publication, methods, country/territories effected by smoke, proximity to inhabited regions, physical effects of bushfire smoke, mental health effects of bushfire smoke, other health outcomes of bushfire smoke, communication strategies, and main results of each paper.

## 3. Results

Sixty-seven studies were included with twenty of the studies based in Australia. Of the remaining forty-seven articles, thirty-two were from the United States of America, eight from Canada, two were global studies, and one study originated from Southeast Asia, the United Kingdom, Portugal, Sri Lanka, Belgium, and China, respectively. Forty papers were found that provided information pertinent to communication and public action during natural disasters. Twenty-one publications relate to the origin and media form, with thirteen providing information on traditional media [9,10,11,12,13,14,15,16,17,18,19,20,21], eleven on non-traditional sources [10,11,15,20,21,22,23,24,25,26,27], and seven on choice of media [9,10,11,15,18,28,29]. Five articles provided information on smoke avoidance behaviours [19,20,22,25,30,31]. Twenty-nine papers provide advice on message style, with nine relating to message content [9,18,20,22,27,32,33,34,35], nineteen relating to message style [11,14,15,18,19,21,22,30,32,33,34,36,37,38,39,40,41,42,43,44], nine relating to the origin of the message [10,14,15,18,21,22,34,38,42,45], and seven specifically mentioning two-way dialogue [15,18,33,36,46,47,48]. Finally, six papers mention communication limitations [10,13,15,18,37,43]. Article summaries can be viewed in Supplementary Materials, Table S3.

Thirty papers reported on elements of adverse health outcomes related to bushfire smoke exposure. Fifteen articles described community medical/health seeking behaviour during bushfire smoke events [49,50,51,52,53,54,55,56,57,58,59,60,61,62,63]. Nine articles documented mortality statistics during these events [51,52,53,54,58,59,63,64,65]. Twelve articles outlined morbidity data [49,50,51,52,53,54,57,58,61,62,66,67]. Eight articles described the psychological impacts of bushfire smoke events [52,53,68,69,70,71,72,73]. Twelve articles highlighted at-risk populations during bushfire smoke events [31,51,52,53,54,57,58,59,61,62,65,74]. Four studies documented in-vitro or animal model studies exploring health consequences of bushfire smoke exposure [53,55,61,75]. Two articles covered both health communication and health effects of bushfire smoke exposure [31,32,33,34,35,36,37,38,39,40,41]. Article summaries can be viewed in Supplementary Materials, Table S3.

### 3.1. Communication Media

Sources of information such as television, radio channels, newspapers, telephone hotlines, community meetings, and websites are heavily used by local and national government bodies tasked with disseminating disaster information, and media organisations to communicate during disasters [10,11,13,14,15,17,18,19,20,21]. Word-of-mouth information is also popular across cultures and geographical location; allowing transfer of relevant information that may not be suitable for mass media broadcast despite the accuracy of transferred information varying widely [9,18]. Of these traditional sources of information, radio (especially local radio) has been emphasised as having a particularly vital role to play in dissemination of disaster information, emotional support, and practical advice in rural and remote settings [12,16]. Local radio is viewed as being both reliable and trustworthy, provided it remains functional during disasters [18].

Social media has become increasingly popular among many levels of government, private organisations, and members of the public during disasters [11,15,20,23,26]. Authorities are using Facebook and Twitter to share short posts containing updates and/or embedded links to government webpages with further information in real-time [11,20,21,23]. State governments have also utilised real-time information provided by Twitter in combination with satellite imaging to develop applications that track bushfires and release warnings to relevant parties [27]. Specialised social media services have also been developed to provide disaster-specific information to at-risk populations [24]. Application engagement appears to be directly motivated by exposure to bushfire smoke [25]. Emergency response organisations receive engagement from the public during natural disasters, with up to 45% of relevant tweets being retweeted by members of the public [22,27]. Public engagement and trust are also influenced by the age of the Twitter account, with older accounts being perceived as being more trustworthy than newly created accounts [22]. Government accounts tend to act as an initiator for information and disproving false news, while relying on individuals to then further disseminate the information [10,22]. Despite this, it should be noted that a large portion of disaster information dissemination still originates from independent members of the public, academic bodies, and voluntary organisations [15,22].

Both Australian and international studies have identified trends in communication preferences during natural disasters. People below the age of 40 are more likely to seek information from social media sites, television, and local newspapers while those above the age of 40 show preference for local radio and newspapers [9,11]. It should be noted that the age of 40 is arbitrary, and likely reflects the age of individuals at which social media platforms gained popularity and so may change in future. Word-of-mouth information is common regardless of age, culture, and country [9]. Rurally located households are more likely to rely on television for disaster information, with less information being received from newspaper and radio sources [9,18,29]. Despite this utilisation of traditional media, households are still likely to have internet access through mobile phones and do receive disaster information from these devices to varying degrees [29].

Preference for media sources has also been found to fluctuate as disasters progress [15]. Prior to a disaster occurring, there is little use of social media sites to discuss emergency information [15]. During disasters, social media platforms have been found to dominate disaster communication channels with content such as warnings, imagery, help requests, health communication, coordination of aid relief, and communication of safety status [15]. When alerting communities of the need to evacuate, most reports came through telephone calls, with other media forms including television, radio, door-to-door knocks, and word-of-mouth [10,28]. Reverse emergency calls were found to be useful in the United States of America as emergency organisations were able to contact residents who may be in danger more directly [28]. Conversely, individuals tend to receive reports from television, radio, or social media when it is safe to return to their residences and when learning of property destruction [10,28]. Newspapers tend to become more relevant and popular as disasters progress as they allow for more in-depth analytic coverage [18]. Facebook and Twitter continue to play a significant role in many countries during the post-disaster phase with organisation of recovery and clean-up operations commonly occurring on the social media platforms [10,15].

### 3.2. Optimising Disaster Communication

Nine studies have identified key elements of communication content that can improve understanding and uptake of crucial information [18,34]. Guidance, timeframes, geographical location, identification of specific hazard, and information source have been suggested as important factors to include [27,33,34]. Providing local context to communications has also been promoted by multiple sources [9,18]. Successful messaging commonly contained information for at-risk populations such as elderly, paediatric, culturally, and linguistically diverse (CALD) and those with pre-existing conditions such as asthma, chronic obstructive pulmonary disease (COPD), and cardiac conditions [20,32,35]. Most communications provide updates rather than directives; although provision of relevant information has been suggested so individuals can make informed decisions [22]. Source hyperlinks have not been definitively proven to increase trust in information provided by public health and emergency service agencies during bushfires/natural disasters [34].

A common suggestion presented by seventeen papers containing communication advice was that use of clear, specific, accurate, certain, and consistent language promoted optimal communication [15,34]. Language terms such as ‘evacuate’, ‘now’, and ‘update’ increase public participation and uptake of messages [19,21,30,34,38,41,43]. Information should be released in a timely manner and be as unbiased as possible [14,15,18,34,37,40]. Using language that evokes fear or panic is not recommended [36]. The reviewed literature also suggested that communications aim to be culturally appropriate and available to impaired populations [18,32,42,43]. Consistent messaging across time and between sources also promotes trust in the information provided [11,14,22,33,34,39,41,44].

The origin of the communication also influences the effectiveness of communication [18,34,45]. Communications should be distributed through as many media forms (including in-person) as possible to ensure maximum delivery to the public, especially in regions that do not regularly experience disasters [14,38,42]. Communications that originate from social media accounts with more followers are more likely to be shared with other members of the public, further amplifying the effectiveness of the message content [34]. Re-tweeting or re-sharing of these messages by other official accounts also increases trust in the message content [18,21,45]. Official accounts should use clear branding that emphasises their official logos, encouraging easy recognition of trusted sources [10,15].

Although most reviewed papers focused on message content, there was also a persistent theme of recommending a dialogue to exist between authorities and the public that allows relevant questions to be answered when possible [18,48]. Repetition has also been shown to increase retention of advice [46]. Recommendations also suggest that local governments and government agencies utilise social media websites and other forms of communication to provide pre-disaster information to the public, aiming to increase disaster literacy and increase chances of relevant information being shared through the public [15,18,33,36]. Logistical planning should occur prior to disasters occurring in order to facilitate a smooth communication process [47]. Acting before disaster occurs could also allow for more flexibility in how disaster information is provided and ultimately improve disaster outcomes [18].

### 3.3. Communication Limitations

Only one study considered deaf and hard-of-hearing populations, finding that appropriate information was rarely available despite poorer health outcomes being common in this population [43]. No studies found information provided for visually impaired populations. Traditional media sources have been criticised for ‘sensationalising’ emergency events, leading to distrust in presented information [18]. Radio information in Australia is also limited by a lack of national service, jurisdictional boundaries, and rules relating to information delivery [13]. Misleading information is also common in social media news, with ‘fake news’ and misleading information being recognised frequently [15]. Structural communication failures also occur, with power outages, website failures, and overwhelmed call centres complicating information delivery [10,18,37].

### 3.4. Medical Admissions

There is suggestive evidence of an association between bushfire smoke exposure and increased general physician attendance rates uniformly across all age groups [76]. Many age groups reflected a statistically significant increase in appointments but inconsistency exists between studies [50,58]. Although asthma was initially hypothesised as being a cause of increased general physician visits after acute bushfire smoke exposure, no studies have presented quantitative evidence to support this [53,63].

Emergency department (ED) presentations [41,50,54,56,60,61] and subsequent hospital admission rates [49,51,52,58,61] increase during bushfire smoke events and in the days following [50]. Most ED presentations on these days relate to asthma, COPD, or non-traumatic conditions [56,60]. Asthma sufferers have an increased relative risk for ED presentation on the day of smoke events for all ages (becoming more significant as age increases) [50]. Despite an increased relative risk, paediatric asthma cases remained low with most asthma presentations being in the adult population exposed to bushfire smoke [56,57]. Unplanned hospital admissions during bushfire smoke events were predominantly caused by respiratory conditions; however, it is unclear if this was statistically significant [52,53,58,59,60]. A statistically significant increase in cardiovascular-related hospital admissions existed during bushfire smoke events in females, the elderly and whole population [51]. There was also a non-significant increase in cardiovascular presentations in the days after bushfire smoke events [56].

### 3.5. Biomedical Effects of Bushfire Smoke Exposure

Respiratory morbidity is associated with bushfire smoke exposure [51,57,58]. Exacerbations of asthma or COPD and respiratory tract infections contribute to most morbidity seen during bushfire smoke events [50,51,52,57,61]. As smoke burden increases, studies have shown an increased distribution and use of reliever medications such as salbutamol [50,53,63]. One study failed to prove an association between increasing particulate matter levels during bushfire smoke events and peak expiratory flow rates consistent with worsening asthma symptoms [67].

Cardiovascular morbidity due to bushfire smoke exposure is unclear with less than half of the relevant studies identifying associations [51,52,58,61,66]. Possible associations have been identified relating bushfire smoke exposure and cardiovascular illness; particularly in elderly and female populations [51,52]. A possible link has also been identified between bushfire smoke exposure and out of hospital cardiac arrest in elderly and male populations [51,52,66]. Notably, only studies conducted in the USA demonstrated any statistically significant association [58,61].

A strong positive association between bushfire smoke exposure and all-cause/non-traumatic mortality is well documented [53,58,59,63,65]. When considering specific respiratory and cardiac mortality, results are less significant [53]. Cardiovascular mortality is acknowledged in several studies, with the largest effect existing in elderly populations [52] exposed to higher levels of smoke pollution [52,65]. It is possible that cardiovascular mortality peaks several days after smoke exposure [52,65]. Respiratory-related mortality is inconsistent, with some studies finding no statistically significant association [51,53] while other studies have identified association in COPD sufferers most commonly 24 hours after bushfire smoke exposure [65].

Cellular responses to bushfire smoke exposure have been recognised in animal and in-vitro studies [61,75]. Increased TNF-α concentration, less viable cell populations, oxidative gene expression, and DNA fragmentation have been identified [61,75]. Inflammation and cytotoxicity have also been presented as bushfire smoke outcomes [51]. It should be noted that study design, cell population, and exposure times varied greatly in studies, possibly contributing to the heterogeneity in current published data [75].

### 3.6. Psychosocial and Mental Health Effects

Seasonal bushfire smoke exposure is usually associated with mild psychological distress [52]. More extreme events involving the threat of fire may foster the development of severe distress characterised by intense fear and uncertainty or contribute to the development of psychiatric illness [52,53,69]. The common precipitating factors for psychological distress during bushfire smoke events include feelings of isolation while sheltering inside from smoke, worsening symptoms of chronic mental illness, and the desire to engage with the outside environment [72]. Distress relating to fire uncertainty, the need to evacuate, and the safety of friends and family is also likely to be the causative factor of psychological symptoms as smoke exposure; with many individuals likely to experience a combination of these stressors [68,71,72]. Communal coping events (commonly held by evacuees [68]) have been identified as valuable, with up to half of those involved in serious bushfire events [53] (more commonly non-evacuees [68]) likely to experience psychiatric illnesses such as depression and post-traumatic stress disorder up to five years after the bushfire event [69].

### 3.7. At-Risk Populations

Adverse outcomes from smoke exposure are more likely in at-risk populations. At-risk populations include paediatric, elderly, and female populations (particularly those who are pregnant) [31,51,52,53,54,58,59,61,74]. Individuals with pre-existing health conditions (respiratory, cardiovascular, and smoking history) and low socioeconomic groups also have reduced health outcomes from bushfire smoke exposure [51,53,54,57,59,61,74]. First Nations people in Australia and people of African or Hispanic descent in the United States have been found to have comparatively worse health outcomes [51,58,65,74]. Uncertainty exists surrounding the effects of education and geographical location on the health outcomes of the general population during and after bushfire smoke exposure [57,59].

### 3.8. Smoke Avoidance Behaviours

Six studies yielded information relevant to smoke avoidance behaviours. Commonly suggested avoidance behaviours were that of activity change (cancellation/relocation of outdoor events), keeping windows and doors closed, and staying indoors [19,20,25,30,31]. Use of N95 masks, home air conditioning, and high-efficiency particulate air (HEPA) filters were less likely to be suggested to the public and were less commonly utilised avoidance behaviours [19,20,25,30]. Evacuation was the least likely intervention to be suggested to all groups and was found to be the least commonly implemented behaviour change [20,25].

When implementing behaviour changes, individuals were more likely to utilise multiple avoidance behaviours rather than singular behaviours [25]. Implementation of avoidance behaviours is positively correlated with a number of smoke exposure symptoms experienced by individuals, as is the likelihood of individuals utilising medication and healthcare services [25]. As the length of bushfire smoke exposure increases, individuals become more likely to implement multiple smoke avoidance behaviours [19]. Individuals with pre-existing medical conditions that necessitated increased inhaler or oxygen use were also found to be more likely to seek follow up medical care and utilise N95 masks during and after bushfire events than healthy populations [20,30,31].

Other predictors for increased adherence to avoidance behaviours include higher education levels, female gender, increasing age, personal concern, frequent health messaging, and having a disability [30,31]. When investigating reasons for non-adherence, lack of time, confusion around message meaning, and pressure to carry out normal activities were common [30]. Caucasian individuals were also less likely to implement behaviour changes, with homeless individuals also being noted to have many barriers impacting on their ability to implement smoke avoidance behaviours [20,30].

## 4. Discussion

This scoping review focused on two inter-linked themes of health communication and adverse health effects of smoke exposure in the context of bushfire smoke events. A total of 67 full-text articles were included in this review. Thirty articles investigated health outcomes following bushfire smoke exposure. Forty articles provided information related to communication techniques and the strategies which may assist in mitigating potential excess morbidity and mortality during bushfire smoke events. Two articles discussed both health outcomes and communication techniques.

### 4.1. Communication during Bushfires

Communities were shown to use a combination of traditional media sources (television, radio, newspapers, and telephone calls) and non-traditional media sources (Twitter and Facebook) when receiving disaster information [10,11,12,13,14,15,16,17,18,19,20,21]. Social media allows for a real-time dialogue to exist between authorities providing information and the public, a concept that has been reviewed favourably in the literature [18,48]. Despite the rise of social media platforms in recent years, there is still high utilisation of traditional media forms (especially radio); highlighting the importance of continuing to use traditional media alongside non-traditional sources in future, to ensure that all aspects of the community are catered for [16]. Different media are also utilised at different stages of natural disasters, indicating the importance of tailoring methods of information delivery to each individual disaster as they progress to their conclusion [10,15,18,28]. Particular emphasis was placed on providing information before a disaster occurs [15,18,33,36]. There has also been scarce implementation of specialised programs, which are custom made for specific natural disasters like bushfires [24,27]. These programs have been reviewed favourably and could be considered on a larger scale in the future to assist with the timely delivery of information to the public.

When reacting to bushfire smoke advice, individuals are most likely to enact multiple actions. Due to the requirement for mask use in the COVID-19 pandemic, future studies could see a rise in mask use in response to poor air quality due to mask use becoming more mainstream.

The content of disaster communication directly influences the effectiveness of the message intended to be shared [34]. Effective communication includes guidance, a timeframe, a location, a hazard or consequence, and a source from which the information came [27,34]. The communication must guide the public toward actions that prioritise their health and safety during the disaster [34]. When possible, information should be tailored to the local context, while ensuring that sources of information always remain credible and consistent across administrative borders [5,9,18]. Clear timeframes should be outlined to guide people’s actions during the disaster [34]. The affected location should be identified, ensuring that those in potentially affected areas are made aware of their status [33,34]. Evacuation sites, smoke and fire affected roads, and closed regions can also be identified [27]. The disaster and related hazards should be described in a manner that highlights the potential effects on health and wellbeing [34]. Finally, the source of the provided information should be clearly included for those wishing for further information, although inclusion of source hyperlinks does not necessarily increase engagement [18,22,34].

Messages should be clear, specific, accurate, certain, and consistent [15,34]. This includes culturally safe messaging that is available in a variety of languages and formats [18,32,42,43]. Clear and simplified language that is free of jargon was advocated by many articles, with terms such as ‘evacuate’, ‘now’, and ‘update’ increasing public participation [19,21,30,34,38,41,43]. In addition, articles noted that it is important to choose language that does not inspire fear or panic [36]. Accurate messaging is achieved by ensuring that communications are shared in a timely manner and provide complete, unbiased, and factual information [14,15,18,34,37]. Messages need to be consistent over time (explaining changes from prior messaging) and administrative boundaries, and avoid conflict of information [5,33,34]. In instances of uncertainty, an authoritative and confident messaging campaign can be used to encourage public confidence [34]. Consistent messaging between information sources also promotes uptake of message content and reduces confusion and distrust [11,14,22,39,41,44].

There are limited communication resources available for at-risk populations like those with disabilities and CALD populations [43]. There were no specific resources presented for paediatric populations. The information suggested to these subgroups is very similar to the information provided to the wider population [20,32,35]. This is despite possibly requiring specific message delivery modes based on audio-visual disability and language proficiency, or requiring additional means to enact health behaviour advice like organised transport services or mobility assistance. Other at-risk groups who may benefit from tailored health messaging are those that are likely to suffer from the adverse effects of being exposed to bushfire smoke. Populations who are likely to be most sensitive include those with pre-existing chronic illness of respiratory and cardiac origin, and First Nations people, those at the extreme ends of age, and those who are pregnant [31,51,52,53,54,58,59,61,74]. The provision of further resources for these subgroups has the potential to improve health outcomes and health literacy within the population [32].

### 4.2. Health Effects of Bushfire Smoke Exposure

ED presentations are more common after bushfire smoke events and these mostly consist of acute exacerbations of chronic respiratory illnesses [56,60,74]. The absence of a proportional rise in hospital admissions suggests that the bulk of these excess cases can be managed successfully without hospital admission [53]. Understanding the presentation patterns during bushfire events could allow medical services to prepare for anticipated healthcare presentation surges. This may involve ensuring adequate provision of staffing and resources, and prompting healthcare workers to recognise potentially smoke-induced acute and chronic disease earlier.

Respiratory illnesses exhibit the strongest association with bushfire smoke-associated excess morbidity based on hospital and emergency department visit rates [56,60,63,74]. This hypothesis is also supported by increased utilisation of bronchodilator medications during bushfire smoke events [53,63,76]. Directing timely preventative health information towards at-risk groups with underlying respiratory disease may yield some improvements in health outcomes during bushfire smoke events [53].

Increased mortality may be associated with bushfire smoke exposure, but heterogeneity exists in the literature [51,52,53,54,58,59,63,64,65]. The effect size associated with excess bushfire smoke event mortality is largest in all-cause and non-traumatic mortality metrics [53,63]. How disease associated with a specific organ system contributes to this excess mortality is not well understood [51,52,53,65]. Despite linking out-of-hospital cardiac arrests to bushfire events [66], other studies have failed to find consistent association between bushfire events and cardiovascular mortality [51]. More research in this area could be of benefit. By better understanding the link between bushfire smoke and cardiovascular disease, preventative health messaging may benefit those with underlying cardiovascular disease.

Psychological distress concurrent with bushfire smoke events is complex in nature. Direct smoke exposure and isolation are implicated as common causes [52,71,72]. While the acute distress of experiencing a bushfire resolves in many, it can result in the development of severe and prolonged psychiatric illness [52,53]. However, the data were mainly qualitative in nature, and it is difficult to differentiate the psychological impacts specifically related to smoke exposure from other potentially traumatising factors like forced evacuation from home, an approaching fire front, or the loss of loved ones and belongings [68,69,71]. There was no specific information or recommendations provided for paediatric populations. Much of the smoke avoidance advice involves isolation indoors and that a degree of distress stems from this action [52,72]. Health messaging could emphasise the value of remaining in contact with loved ones and suggest means to remain engaged while bushfire smoke remains a hazard outside.

### 4.3. Strengths and Limitations

This study has included a significant time span from which to gather articles that document the progression to our current understanding of how bushfire smoke exposure may affect health and the means to best communicate relevant health behaviour information. We have captured international studies over approximately two decades, which explore many major case study bushfires and other natural disasters. This provides novel information about international approaches to public health communication during bushfires as technology rapidly develops over time. 

However, one must consider the date that this paper and its referred communication information studies has been published. With time, older articles in our database are progressively less likely to reflect the growing reliance on communication technology in the global population. One limitation of this study concerns the unpredictable nature of bushfires and the inherent tendency to reactively perform retrospective analyses. Most communication articles lack specific quantitative evidence around pre-fire preparation, which would likely be a key element of any community bushfire preparedness plan. This form of study also precludes the ability to quantify the impact of communication techniques on specific adverse health outcomes. This metric could be used to rationalise employing components of health communication strategy during different phases of the fire and to specific at-risk populations.

The outcomes of this study also reflect the lack of diversity in populations and study locations available in the literature. Most articles failed to consider specific at-risk populations in any significant way, limiting this review from providing detailed findings or recommendations for these groups. Future research focusing on specific at-risk populations could provide valuable insight into management of smoke exposure. For example, CALD groups and those with hearing, vision, and mobility-related disabilities may benefit from work to identify how to best prepare or notify them when bushfire events occur. Many articles included from our literature search are also based in Australia and North American regions. These locations tend to contain populations with similar characteristics in terms of income, spoken language homogeneity, communications modality preferences, and access to advanced technologies. Therefore, recommendations described here may be less reflective of lower/middle income and/or more linguistically/culturally diverse countries in Europe, South America, Asia, and Africa.

## 5. Conclusions

Bushfires are likely to become a bigger public health problem in the future due to the ongoing effects of climate change [7,77]. Bushfire smoke can affect a much larger portion of the population than fire directly, as evidenced by the Australian Black Summer bushfires of 2019–2020 [1]. By communicating health behaviour advice effectively in terms of the timing, the delivery medium used, and the content and style of the message, there is an opportunity to mitigate the health consequences of bushfire smoke. These likely include significant short-term and long-term impacts on human health, especially for at-risk populations and those who have an underlying chronic cardiorespiratory condition, and produce an additional burden on healthcare services [49,50,51,53,58]. This paper presents a scoping review of the literature upon which to base further investigation and inform public health policy about health communication during bushfire smoke events. Quantitative comparison of communication methods may yield more specific recommendations for health messaging in future bushfire smoke events.

## Supplementary Materials

The following are available online at https://www.mdpi.com/article/10.3390/ijerph182010889/s1. Table S1: Search terms used for the PubMed, ProQuest and Web of Science databases. Table S2: Inclusion and exclusion criteria used in the scoping review. Table S3: The articles included in this scoping review and their key objectives and findings.
