# Supplementary material for: Efficacy of Communication Techniques and Health Outcomes of Bushfire Smoke Exposure: A Scoping Review
Source: Int J Environ Res Public Health. 2021 Oct 16;18(20):10889. doi: 10.3390/ijerph182010889 (PMC8536189; doi:10.3390/ijerph182010889)
Supplement: Supplementary file 1 [file ijerph-18-10889-s001.zip › ijerph-1390695-SI.pdf]

International Journal of
Environmental Research and Public Health

MDPI

Efficacy of Communication Techniques and Health Outcomes of Bushfire Smoke Exposure: A Scoping Review

Emily Heaney [1], Laura Hunter [1], Angus Clulow [1], Devin Bowles[2], Sotiris Vardoulakis[2]*

[1] ANU Medical School, Australian National University, Canberra, ACT 2601, Australia
[2] National Centre for Epidemiology and Population Health, Research School of Population Health, Australian National University, Canberra, ACT 2601, Australia
* Correspondence: Sotiris.Vardoulakis@anu.edu.au

Supplementary Materials

Table S1: Search terms used for the PubMed, ProQuest and Web of Science databases

PubMed database	ProQuest database	Web of Science
Bushfire	Bushfire	Bushfire
Landscape fire	Wildfire	Wildfire
Biomass open fire	Smoke event	Smoke event
Peat fire	Health communication	Forest fire
Prescribed burn	Health communications	Health communicat*
Risk reduction burn	Health message	Health messag*
Wildfire	Health messages	Social media
Wildland Fires	Information dissemination	
Brush Fires		
Brush Fire		
Fire, Brush		
Fires, Brush		
Forest Fires		
Fire, Forest		
Fires, Forest		
Forest Fire		
Wild Fires		
Fire, Wild		
Fires, Wild		

Wild Fire<br>Health messag*<br>Communicat*<br>Warning<br>Fact sheet<br>Guid*<br>Info*<br>Updat*<br>Social media<br>Media, Social<br>Social Medium<br>Twitter Messaging<br>Messaging, Twitter<br>Web 2.0<br>2.0s, Web<br>Web 2.0s		

Table S2: Inclusion and exclusion criteria used in the scoping review

Inclusion criteria	Exclusion criteria
• Human smoke exposure.<br>• Any location – worldwide, urban/rural.<br>• Communication strategies that focus on reducing risk of exposure/adverse health outcomes prior to, during and after fires.<br>• Reduced disease morbidity or mortality related to smoke exposure.<br>• Increased disease morbidity or mortality related to smoke exposure.<br>• Quality of life outcomes post bushfire smoke exposure.	• Focus on outcomes related to non-bushfire smoke – industrial fires, household fires, grass fires etc.<br>• Health outcomes for emergency services – firefighters, police, paramedics.<br>• Papers not written in English.<br>• Occupational exposure to smoke.

Table S3: The articles included in this scoping review and their key objectives and findings

Author	Design	Country	Main objectives	Key findings
**Abedin et al. (2018) [22]**	(Retrospective) time series	Australia	To investigate the role of individual Twitter accounts in disaster communication dissemination when compared to formal sources.	Both individuals and large organisations used Twitter to distribute fire-related information. Content is typically informative rather than directive.
**Afifi et al. (2012) [68]**	Retrospective cohort study	USA	To investigate the impact of disasters on community uncertainty and how communal coping affects recovery.	Evacuated vs non-evacuated persons differed on every variable. Variables included uncertainty about homes/loved ones, reliance on communal coping, and levels of stress. Communal coping did not change concern levels relating to home, self, or others. Age and sex varied the experience of mental illness. Older women had less distress compared to other age groups.
**Anderson-Berry et al. (2018) [36]**	Review	Australia	To summarise current warning-based systems and investigate their potential use in Australian natural disasters.	The study recommends the development of a warning system that encapsulated warnings, possible impacts, relevant information, and research for common natural disasters in Australia. Definition of 'warning' was poorly understood by stakeholders. Case studies highlighted that inaction/inadequate action is linked to poor risk perception. Messages must communicate risk to prompt action while promoting self-efficacy. Warning systems are more effective when communities are pre-prepared for disasters.
**Andrulis et al. (2011) [32]**	Mixed methods - systematic review, qualitative interviews	USA	To identify challenges and barriers in reaching ethnically diverse communities prior to, during and after disasters. To highlight possible policy strategies and areas for future development and research.	Individual-level barriers included culture, language and socioeconomic status while institutional-level barriers included inadequate support for cultural and linguistic initiatives. Increasing public-private partnerships, language assistance services, engagement strategies, collaboration across sectors, and capacity for diversity initiatives could improve these barriers.
**Bearman et al. (2015) [37]**	Qualitative thematic study	Australia	To use case studies to identify and investigate decision-making breakdowns and the effects of these breakdowns at management team levels and above.	Decision breakdowns included informational (parties had different information), evaluation (information interpreted differently), and operational (parties had different views on how information should be acted upon). Disconnects were the most common breakdown, however multiple disconnects commonly occurred sequentially to result in major breakdowns. Improved management was seen during technical operations when compared to unplanned fires, thought to be due to predictability.

**Beggs et al. (2019) [64]**	Literature review	Australia	To review data on the consequences of climate change relating to human health.	Increasing fire severity, duration and size led to more significant adverse outcomes such as economic loss, property destruction and loss of life. Projected increases in air pollution will adversely affect health outcomes. Premature mortality caused by bushfire smoke exposure was expected to double by 2100. Four-year averaged person-day smoke exposure rates increased from 249,397 to 266,744 when comparing 2001-2004 and 2015-2018 in Australia. Both of these numbers could result in substantial adverse outcomes.
**Bernstein et al. (2013) [49]**	Expert commentary	USA	To comment on the effect of climate change on respiratory morbidity in those with chronic lung diseases.	Climate change increase bushfire incidence, subsequently increasing air pollution over large areas. Air pollutants included particulate matter (PM), carcinogens (formaldehyde, benzene) and acrolein. Bushfire smoke was linked to increases in emergency department presentations, hospitalisations and asthma exacerbations.
**Borchers-Arriagada et al. (2019) [50]**	Systematic review and metanalysis	Australia	To perform a systematic review and meta-analysis on short-term exposure to wildfire smoke PM2.5 and asthma outcomes.	Increased emergency department presentations occurred on the same day and the day following elevated $PM_{2.5}$ levels. Females, adults, and elderly populations more frequently presented. Significant association between bushfire smoke and salbutamol dispensation was reported.
**Brengarth et al. (2016) [23]**	Qualitative – case study	USA	To investigate the role that Web 2.0 applications (communication platforms, social trends, and business strategies) have on crisis communication and disaster situations.	Web 2.0 applications provided flexible methods of information sharing in real-time during disasters. Applications clarified information and connected parties in a timely manner while preventing the spread of false information. Non-profit organisations and the general public could minimise first-responder burden, divert 911 calls, fundraise and deliver support materials to responders.
**Bryant et al. (2018) [69]**	Longitudinal cohort study	Australia	To examine the psychological outcomes experienced by communities affected by the Black Saturday Bushfires 5 years post-event.	Fire-related post-traumatic stress disorder, major depression, alcohol misuse, and other mental illness reduced over time. Psychological distress remained high, suggesting that life stressors may trigger this distress and result in long-lasting effects.
**Burns et al. (2010) [9]**	Questionnaire and thematic analysis	Australia	To investigate the understanding of bushfire emergencies and communication strategies by different community groups.	Those over forty years of age had the most bushfire experience, were more likely to be members of local organisations, and used several information sources but preferred trusted local sources such as emergency services and councils. Younger people utilised television, local newspapers and word-of-mouth. Young parents had less trust in government sources and lacked specific local knowledge. All ages desired clear, timely and specific information directed to local conditions and communities.

**Cascio (2018) [51]**	Literature review	USA	To investigate the health effects of wildfire smoke exposure and identify the role of healthcare professionals in mitigating the adverse effects of wildfire smoke exposure.	There was a positive correlation between bushfire smoke exposure, $PM_{2.5}$ levels and all-cause mortality. Respiratory morbidity from asthma, pneumonia and chronic obstructive pulmonary disease was also linked. Results from cardiovascular morbidity and mortality was mixed. At risk populations included elderly and paediatric populations, pregnant persons, middle-aged adults, and those with underlying cardiorespiratory disease.
**Chauhan et al. (2014) [10]**	Retrospective review	USA	To examine the timelines, behaviours and contributions of official online information during the 2014 Carlton Complex wildfire.	The most relevant and timely information related to bushfires, evacuation, and fire destruction was provided by local news media. More reliance was placed on local media than first responder agencies. Event-based resources came directly or indirectly from official emergency response agencies. Some of these agencies continued to provide information post-disaster, highlighting the needs of affected communities. High volumes of relevant information was provided but the source of information was unclear at times. Clear identification of agencies could improve credibility and community trust.
**Cheong et al. (2019) [52]**	Scoping review	South-East Asia	To determine the existing knowledge of the public health impact of South-East Asian transboundary haze.	Broad categories of health impacts from smoke exposure were reported. These included sore and dry throat, nose and eye discomfort, headache, shortness of breath, and skin irritation. However, the extent of health impacts that were not associated with underlying illness was unclear. All impacts were linked to mild distress with a possible psychosomatic component.<br><br>Respiratory diseases had a dose-dependent relationship with smoke exposure, with upper and lower respiratory infections, rhinitis, and decreased lung function being linked. Elderly and very young patients were at the highest risk of respiratory illness. There was also increased hospital admissions for those with chronic obstructive pulmonary disease and asthma. Neurological impacts (stroke, migraine) were less clearly linked with smoke exposure and could be due to underlying cerebrovascular illness. Cardiovascular illness (myocardial ischemia, cardiac arrest) incidence was increased, however the mechanism of this was unclear and could be directly or indirectly related to PM toxicity, systemic inflammation, oxidative stress, or accelerated atherosclerosis. Cardiovascular illness lagged behind smoke exposure, reaching highest levels 2-5 days post-exposure and showed highest mortality in elderly and paediatric populations.
**Corrieri et al. (2019) [62]**	Expert opinion summary post conference	USA	To discuss and evaluate the impact of wildfires on agriculture and professionals working in	Smoke exposure could result in hospital admissions and cardiorespiratory complications. Other consequences include heat exhaustion, stroke and mental illness (anxiety, depression, post-traumatic stress disorder). At risk groups include

			agricultural and occupational health.	children, pregnant persons, and those with chronic cardiorespiratory illness. Air quality indexes should monitor hazard levels and water quality.
**Cox et al. (2011) [71]**	Ethnographic, mixed methods	Canada	To determine the effect of place, identity, and social capital on community resilience and the disaster recovery process.	Disaster recovery should include the role of place and recognise how disasters disorientate and disrupt individuals. A reorientation process should be developed that integrates social-psychological response to better understand and adapt recovery processes.
**Damon et al. (2010) [38]**	Expert opinion summary post conference	USA	To identify the most effective public health response to smoke events associated with wildfires.	Behaviour change was achieved by providing new communications to overcome the increasing frequency on planned and unplanned smoke events. Messaging should aim to communicate the threat, community susceptibility and desired actions. Air quality data should be reported regularly to achieve appropriate responses. Communications should be tailored to each channel where possible, or a generic message can be released in the context of insufficiently trained staff. Messaging should be flexible and reactive to weather changes.
**D-Antoni et al. (2017) [30]**	Systematic review with narrative description	UK	To understand how air quality warning systems influence protective behaviours and identify demographic and psychosocial factors that influence adherence to health advice.	Protective behaviours included rescheduling outdoor activities, avoidance of busy roads and taking preventative medications. Reducing/rescheduling outdoor activities had the lowest adherence rate. Adherence rates were improved by knowledge of where to check air quality and health advice. Barriers to adherence include poor understanding of air quality indices, lack of time, reliance on sensory cues, and exposure to information that reduced concerns of air pollution.
**Dennekamp et al. (2015) [66]**	Case-control study	Australia	To measure the association between bushfire smoke exposure and out of hospital cardiac arrest in cities during a severe bushfire season.	Higher association between out of hospital cardiac arrest and air pollution levels during bushfire season existed for males but not females. 23.9 excess arrests occurred in the community due to elevated $PM_{2.5}$ during 2006-2007 (a total of 2046 arrests during this period).
**Dodd et al. (2018) [72]**	Qualitative – semi-structured interviews and qualitative analysis	Canada	To explore the impact of bushfire smoke on the mental, emotional, physical health and wellbeing of First Nation communities affected by the 2014 North Western Territories bushfire season.	Mental and emotional wellbeing were influenced by experiences of evacuation, isolation, fear, stress, and uncertainty. Inability to work also adversely affected mental, emotional and physical wellbeing. A link exists between prolonged smoke events, time spent indoors and respiratory illness.
**Dong et al. (2017) [75]**	Literature review	Australia	To identify knowledge gaps relating to the toxicity of bushfire smoke in cell lines.	$PM_{2.5}$ was the major pollutant in in-vitro assessments; with cytotoxicity determined by waste accumulation. Substantial variation existed between studies based on cell types, PM extraction methods, exposure concentration, and timing. Negative effects were seen in all cells. Smaller particles produced worse outcomes compared to

				larger particles. Bushfire PM was more toxic than ambient PM. Bushfire PM appeared to involve different mechanisms – TNF-α, reduced cell viability, more reactive oxygen species production, DNA damage, oxidative gene expression, and increases in cytotoxic markers. Exposure to woodsmoke PM can result in the acceleration of cell apoptosis, oxidative stress, and DNA fragmentation.
**Doubleday et al. (2020) [65]**	Case-crossover study	USA	To examine the association between non-traumatic mortality and bushfire smoke in Washington State.	Non-traumatic mortality was greater on bushfire smoke event days and on the days after smoke exposure in all age groups. Respiratory mortality was raised on the same day of bushfire smoke exposure. The odds of mortality in the days post-exposure to bushfire smoke was also higher than when compared to non-bushfire smoke event days.
**Ekundayo et al. (2018) [39]**	Literature review and case studies	USA	To identify different strategies to improve communication between relief organisations and communities during emergencies.	Coordination failures between emergency services and public/private organisations could lead to ineffective responses. Organisations typically worked independently, resulting in poor integration during disasters. Increased integration, sharing of information, and adoption of new technologies (social media) to integrate structured and unstructured data could improve emergency response. Sustained collection and monitoring of data also improved response.
**Felix et al. (2015) [73]**	Semi-structured interviews, questionnaires and thematic analysis	USA	To investigate the effects of wildfires on family functioning and post-traumatic growth.	Young people, females, and those using positive reappraisal coping had higher levels of post-traumatic growth (PTG), higher fire stress, and life stressors. PTG was measured in families as a group and in young people individually. Positive correlations existed between PTG expressed in families and perceived fire stress and family type. Positive re-appraisal and coping was positively correlated to improved family functioning.
**Finlay et al. (2012)[53]**	Literature review	USA	To collate and synthesise global data relevant to the effect of wildfires on human health.	Wildfires can severely impact health with certain subpopulation groups at higher risk as wood smoke contains high levels of toxins and PM. Communication with affected populations was essential in reducing risk for health impacts. Respiratory disease was the most common health impact. However, cardiovascular disease, ophthalmic disease, and psychiatric illness were also reported.
**Fish et al. (2017) [11]**	Systematic review	Australia	To collate international post-2009 evidence relevant to effective health messaging during smoke events.	Many different media channels were used to disseminate smoke-related communications. However, there was limited evidence on their effectiveness for subpopulations or communities. Messages were more likely to be recalled, understood, and complied with if simple language was used. Socioeconomic status

				affected compliance. At-risk groups were more likely to be told to stay inside relative to the general public to reduce health risk.
**Haikerwal et al. (2015) [54]**	Literature review and case study	Australia	To investigate how smoke generated from prescribed burns effects air quality, PM levels and health.	Prescribed burns manage fuel loads and reduce severity of wildfires. Prescribed burns could majorly contribute to local air pollution despite being smaller scale than wildfires. Prescribed burn smoke is considered a public health concern due to the known side effects of wildfire smoke sourced $PM_{2.5}$ exposure. More research is required to quantify health effects and devise appropriate evidence-based interventions for risk mitigation.
**Hano et al. (2019) [46]**	Structured interviews, thematic analysis	USA	To investigate the association between cardiovascular disease and knowledge of air quality in a cardiac rehabilitation clinic	Air pollution may be an unrecognised risk factor for cardiac patients. Cardiac rehabilitation offers the optimal setting to communicate health risks associated with air pollution. Existing knowledge and behaviours should be considered and integrated into messages.
**Hesterberg et al. (2009) [55]**	Literature review	USA	Aims to investigate the effect of NO_2 short-term exposures on respiratory emergency presentations and hospital admissions.	Healthy individuals exposed to NO_2 below 1ppm did not show inflammatory response. Increased susceptibility to viral illness was also not consistently proven. Exposure to 2ppm for four hours showed increases in neutrophils and cytokines in bronchial lavage specimens but did not necessarily correlate with prolonged or significant lung function changes. In chronic respiratory disease sufferers, inflammation was not expected below 0.6ppm, however one research group showed increased pro-inflammatory processes at 0.26ppm. NO_2-induced AHR studies suggested that asthmatics were unresponsive to concentrations up to 0.6ppm, although some subjects reacted to concentrations as low as 0.2ppm. Extra-pulmonary effects required NO_2 concentrations above 1-2ppm.
**Hugelius et al. (2019) [12]**	Literature review	Various	To investigate the use and the effect of humanitarian radio in disaster health interventions.	Provision of health information and psychological support through humanitarian radio could promote physical and psychological wellbeing during natural disasters. Community engagement promoted resilience, self-efficacy and community efficacy. Radio could also reach large populations in regions with damaged infrastructure in a low-cost manner and contribute to improved recovery and wellbeing of communities and individuals.
**Jalaludin et al. (2000) [67]**	Prospective cohort study	Australia	To investigate how the 1994 Sydney bushfires affected peak expiratory flow rates (PEFR) in children with wheeze.	During the Sydney bushfires, the maximum daily PM_{10} was nearly seven times higher (210 $\mu g/m^3$) than the rest of January and February 1994. There was no significant relationship between PEFR and PM_{10}. Significant negative association was found in children with no bronchial hyper-reactivity. No models revealed significance in the bushfire period.

**Johnston et al. (2014) [56]**	Case-crossover	Australia	To measure the effect of bushfires in Sydney between 1996-2007 on emergency department presentations.	Smoke exposure events were associated with rapid increases in respiratory presentations and delayed presentations in heart failure and ischaemic heart disease presentations. Impacts for those under 15 years old were considerably lower than those older than 15 years old. However, inconsistent associations were drawn between smoke exposure and cardiovascular disease.
**Kain et al. (2020) [40]**	Scoping review, qualitative content analysis	Canada	To present suggestions from family physicians in Canada relating to how public and professional agencies could improve crisis and emergency risk communication.	Suggestions included having a single trusted source for information with concise communication and to consider the needs of those still training in healthcare. In addition, ensuring universal access, and improving the collaboration between public and family medicine, including the development of patient information sheets. Finally, the development of communication infrastructure prior to crises occurring was also suggested.
**Kondo et al. (2019) [57]**	Meta-analysis	USA	To review the effect of bushfire smoke on emergency department presentations and hospital admissions. Focused on the effects on different subpopulations.	Wildfire smoke exhibited greater effect on respiratory health in female chronic obstructive pulmonary disease and asthma sufferers when compared with males. Lower relative risk for all respiratory outcomes was present in young people compared to adults. Data was insufficient to evaluate quantitatively when stratified by ethnicity, income, healthcare access, house occupancy, geography (including urban and rural status).
**Liu et al. (2015) [58]**	Systematic review	USA	To summarise the effects of wildfire smoke on public health, focusing on synthesising existing information and presenting gaps in knowledge.	Measured the degree of wildfire exposure and associated health outcomes was a challenging in current literature due to the difficulty of ascertaining the degree of pollution directly caused by wildfires. This challenge required new methods of differentiating between pollution caused by wildfires and ambient sources. Children, elderly, and those with chronic cardiorespiratory diseases appeared more susceptible. Further research on mortality and cardiovascular morbidity is also required. Development of new methods may clarify wildfire effects and guide mitigation policies.
**Liu et al. (2017) [74]**	Retrospective cohort study	USA	To estimate fine particle exposure from wildfires and subsequent respiratory hospital admissions in subpopulations.	High-pollution smoke exposure was most common in African Americans who lived in urban counties in California. Respiratory admissions were more common in females (10.4% vs. 3.7% in men) and African Americans (21.7% vs. 6.9% in Caucasians) on smoke-wave days when compared to non-smoke-wave days. Non-significant associations were also present in lower-education regions when compared to higher-education regions.
**Liu et al. (2020) [41]**	Semi-structured interview, thematic analysis	USA	To identify features of effective communication from governments and leaders during crises.	Leadership in the context of crisis communication requires crisis perceptiveness, humility, flexibility, and cooperation with various stakeholders.

**Lyth et al. (2018) [31]**	Semi-structured interview, thematic analysis	Australia	To investigate perceived level of health risk, experiences of smoke exposure. To investigate local experiences of risk management and risk communication during prescribed burns/smoke events.	"Place" influenced how information is absorbed and utilised when managing the effects of planned burns. "Place" also affected human agency and community resilience.
**MacIntyre et al. (2019) [42]**	Scoping review, workshop	Canada	To investigate how extreme weather and climate change risk communication informs public health messaging in local regions. To investigate whether risk communication used by these agencies meet Ontario Public Health Standard (OPHS) 2018.	Public health practitioners must utilise effective risk communication which motivates local action and mitigates the effects of extreme weather/climate change to meet the OPHS 2018. Short-term extreme weather events' risk communication was more successful than climate change risk communication.
**Martin et al. (2012)[13]**	Stakeholder submissions and thematic analysis	Australia	To determine the functionality of emergency communication warning systems (ECWS) in Australia.	Many ECWS were active in Australia, however, improvement of disaster management could occur with new ECWS development. This would require redundancy incorporated into current infrastructure. Limitations in current models suggested that benefits could occur with the construction and use of integrated or systems-based approaches.
**McCool et al. (2006) [33]**	Literature review, workshop	USA	To explore the consequences of wildfires in communities and different communication strategies used between organisations and communities using an events-based technique.	Before the fire public awareness should be raised surrounding the role of fire in the ecosystem, the consequences of communities living in this ecosystem, and evacuation procedures. Communication medium, content, and style affected the outcome of communication at this stage. During the fire event communication focused on the logistics of the fire, potential consequences, resource allocation, and required actions of the public (including evacuation). Communication could begin to collect data on public health needs and direct public inquiries to appropriate organisations. After the fire, public interest was highest in relation to fire awareness and planning. Communication was limited by the consistency of information in large fires when communication teams are rotated and replaced, slowing down the passing of critical information.
**Medford-Davis et al. (2014) [47]**	Interview and thematic analysis	USA	To collate expert opinions relating to disaster communication to improve World Health Organisation protocols.	This report recommended the development of public announcements in multiple languages pertaining to various themes. These included: frequently asked questions, maintenance of statistics of types of disasters, different regions, recording sources of trusted local information and contact details of employees of both government and non-government organisations. The report also recommended development of

				a 24-hour worldwide network of participants from every time zone, allowing materials to be updated/accessible at all times.
**Molyneaux et al. (2019) [70]**	Retrospective cohort study	Australia	To investigate self-reported assault/violence in communities affected by the 2009 Victorian bushfires. Also investigates associated links between violence, mental health and alcohol misuse during this time.	Areas which experienced extreme bushfire effects were significantly more likely to report violence when compared to less affected bushfire regions. There was a significant relationship between disaster-effectiveness and violence for women; this was not experienced by men. Women with lower incomes were also more likely to experience violence in highly bushfire affected areas. Women who experienced post-disaster violence were also more likely to experience post-traumatic stress disorder and depression symptoms.
**Mosites et al. (2018) [24]**	Case studies and thematic analysis	USA	To investigate the functional processes of the Local Environmental Observer (LEO) Network through three case studies.	The benefits of LEO included providing a platform that links community members to experts who can advise on food safety, air quality and waste disposal. The system can also indicate community health issues and disseminate information. Current limitations included the bias of self-reporting, uncertainty of diagnosis, poor systemisation, and confidentiality concerns. These limitations did not prevent LEO from acting as an indicator of issues that may require follow-up.
**Muniz-Rodriguez et al. (2020) [48]**	Systematic review	USA	To highlight the public health implications of social media use during emergencies by assessing how social media improves message dissemination and how it identifies the physical/medical/emotional needs of the public.	Social media sites had multiple functions such as broadcasting, sharing public health emergency warnings, and identification of areas requiring relief and medical assistance. Data was visualised using self-reported location and map development.
**Neaves et al. (2014) [28]**	Retrospective cohort study	USA	To evaluate the Reverse 911® system used in the 2007 San Diego fires. Also outlines hazard knowledge, preparedness, and social consequences of evacuations.	Reverse 911® system sent automated messages to individuals’ phones providing warnings and information. 42% of people received first warning calls through Reverse 911® while 7% received the same call after already being warned through another communication channel.
**Neuhauser et al. (2013) [43]**	Literature review, semi-structured interviews, content analysis	USA	To review the availability and readability of literature related to emergency preparedness communications specific to	Few resources exist that were aimed at deaf/hard of hearing (HH) populations. Less than half of the publicly available materials were relevant for deaf/HH populations. Most contained language suitable for 10th grade to university level. Many resources did not provide appropriate graphics, layout or typography. Recommendations

			Deaf/hard of hearing (HH) and elderly populations.	included benchmarks for readability and improvements for emergency preparedness.
**Oliveira et al. (2020) [59]**	Retrospective cohort study	Portugal	To identify the extent of the 2017 forest fires in Portugal, characterise the impact of the fires on PM concentration, and predict potential public health risks of future fires.	PM concentration exceeded European and national air safety guidelines. The fires were also associated with morbidity and mortality. Public health advice should be guided by regular monitoring of PM levels.
**Olsen et al. (2014) [14]**	Semi-structured interviews	USA	To identify public health issues in regions commonly affected by smoke, while summarising the effects of communication between the public and public health agencies.	Common methods of communication included radio, websites, and phone hotlines. Communication challenges included message content inconsistencies, uncertainty of strategic efficacy, and stakeholder goal prioritisation. Recommendations include social media utilisation, coordination of public and inter-organisational communication, and organisation of priorities.
**Prasadi-Kanchana-Jayasekara et al. (2019) [15]**	Retrospective cohort study	Sri Lanka	To characterise the style of content shared in Facebook posts during different stages of a disaster.	Individuals, volunteer groups, and news organisations shared content. Prior to the disaster no content communicating risk was released. Most risk communication was released during the disaster. This included sharing disaster warnings, requesting assistance, sharing contact details, reporting rescue progress, requesting donations, and providing feedback.
**Rappold et al. (2019) [25]**	Prospective cohort study	USA	To assess the value of Smoke Sense, a program that provides information about air quality and subsequent recommended health behaviours.	The demand for information during bushfire smoke events was clear. Individuals did not always recognise their personal risk in disaster situations. Health behaviours were encouraged and more likely to be implemented after symptom onset rather than as a prophylactic measure.
**Robinson et al. (2003) [16]**	Opinion	Australia	To describe the personal experience of a general practitioner during bushfires and their recommendations for future disaster planning.	General practitioners occupied a crucial role that involves direct patient care, referrals, coordination of medical services/essential services and planning post-disaster recovery.
**Ryan et al. (2018) [17]**	Exploratory survey, literature review	Australia	To identify where people found initial danger alerts and subsequent information during flood disasters.	Individuals were most likely alerted to floods via television or radio. Other sources include news/weather websites and social media. Word-of-mouth information was most commonly delivered via phone calls as opposed to text message or in-person communication.

**Sachdeva et al. (2017) [26]**	Retrospective cohort study	USA	To investigate the use of data from social media in the monitoring of consequences from poor air quality during wildfire smoke events.	Communication topics commonly included property damage, impact of smoke pollution, fire features, and concerns for firefighters. Communication topics fluctuated during phases of the fire event.
**Sharp et al. (2013) [44]**	Literature review, semi-structured interviews, content analysis	Australia	To explore issues of trust between the community and governing agencies during a wildfire.	Communication was key throughout all stages. Communication before fires developed trust between communities and governing bodies. Cooperation and acknowledgement of community concerns was vital. Information delivery should avoid broadcast fatigue, but allay feelings of community uncertainty and abandonment.
**Slavkovikj et al. (2014) [27]**	Literature review and commentary	Various	To outline how social media is used in the detection and management of bushfires and other hazards.	Social media such as blogs, Facebook, and Twitter can collect information in real-time in order to identify disaster origins and track disaster development.
**Steelman et al. (2013) [18]**	Semi-structured interviews, thematic analysis	Canada	To identify the major components of effective public health communication during bushfires.	Ideal communication identified was regular, consistent, involved the target audience and provided specific instructions. Planning communications before disasters was optimal as high traffic in call centres and the physical destruction of communication services can pose limitations on effective communication.
**Steelman et al. (2015) [45]**	Mailed surveys and interpretation response	Canada	To describe the disaster experience of community members who receive emergency communications.	Before 2010 information was more often received from television, radio, newspapers, and word-of-mouth. Word-of-mouth sources were most commonly used, but considered less trustworthy sources. Fire departments, forest services and incident management teams were considered trustworthy information sources but were commonly underutilised.
**Sugerman et al. (2012) [19]**	Retrospective cohort study	USA	To identify how residents were informed and their level of understanding and compliance regarding public health communications during bushfire events.	Persons with underlying health conditions were more likely to follow health advice, and were more likely to suffer health complications from bushfire smoke exposure. Most people received information from television with 97.8% being considered understandable. Simple instructions such as 'stay indoors' were more easily recalled. More complex instructions such as utilisation of specific masks was less likely to be actioned. Public health recommendations that were best remembered were more likely to be actioned.
**Sutton et al. (2014) [34]**	Analysis of social media data	USA	To identify which features result in content dissemination among social media users.	Viral Tweets in disaster settings typically included short messages which are limited to a single theme or pair of themes and provide situation updates rather than specific instructions. Tweets were typically difficult to verify and benefitted from the inclusion of links to relevant reference materials.

**Tham et al. (2009) [60]**	Time-series ecological study	Australia	To describe the relationship between the temporal change in air pollution and respiratory disease in a bushfire scenario.	Emergency presentations for children under 15 years increased during bushfire smoke events, but no change in hospital admissions was noted. The strongest contributing factor to this trend after controlling for confounders was PM10 concentrations.
**Vaidyanathan et al. (2018) [35]**	Opinion	USA	To evaluate a tool developed by the Centre for Disease Control which analyses short-term air quality predictions during fires to identify at-risk populations.	The tool has the potential to bolster competency in tailoring smoke event responses to at-risk populations. This may improve time-to-response capacity and expedite the subsequent recovery process.
**VanDeventer et al. (2020) [20]**	Communication content analysis	USA	To analyse government and news media messaging during a bushfire smoke event for effective elements.	Government social media messages commonly contained health risk information and links to parent websites for additional information. They contained risk mitigation or administrative actions less frequently. Media sources were more likely to mention evacuation. At-risk populations such as children, elderly and chronic disease sufferers were encouraged to stay home. No information was provided for homeless populations.
**Wang et al. (2016) [21]**	Communication content analysis	USA	To identify whether social media could be utilised to increase situational awareness during bushfire events.	Twitter content relating to fire activity peaked one day after the fire began. Tweet volume increased in regions nearest to the fire. Common message content mentioned evacuations, safety of personal property, fire location, and public response. Government broadcasts and news media were often re-tweeted.
**Wu et al. (2018) [61]**	Clinical review	Canada	To identify the respiratory and cardiovascular consequences of elevated PM levels.	The inflammatory complications of PM exposure identified could include respiratory disease exacerbation. Correlation with cardiovascular outcomes was not statistically convincing. Susceptible populations included the extremes of age, poorer populations, and those with underlying health problems. Many inflammatory changes were detected in animal studies.
**Xu et al. (2020) [29]**	Retrospective cohort study	China	To determine the relationship between personal experience and exposure to news media during an earthquake.	Rural populations relied more on television media and internet sources on mobile phones. Traditional media exposure did influence disaster experience and perceived risk, reducing the fear of future events and subjective assessment of disaster severity.
**Yao et al. (2020) [63]**	Computer modelling assessment	Canada	To assess amendments to the Air Quality Health Index (AQHI) and their success in estimating health consequences of bushfire smoke.	Amendments best predicted cardiovascular-related physician visits and all-cause mortality during smoke events. The one-hour $PM_{2.5}$ AQHI-Plus amendment best predicted asthma-related outcomes during smoke events.
